# Beyond the grid: Navigating water supply and sanitation service ecosystems in informal settlements

**DOI:** 10.1371/journal.pone.0342657

**Published:** 2026-03-13

**Authors:** Ana Casas, Paul Hutchings, Andrew R. Bell, Beata Kupiec-Teahan, Amy R. Lewis, José Mendoza Sanchez, Simon Willcock, Fiona Anciano, Dani J. Barrington, Mmeli Dube, Caroline Karani, Arturo Llaxacondor, Hellen López, Anna L. Mdee, Alesia D. Ofori, Joy N. Riungu, Kory C. Russel, Bjørn R. Kristensen, Alison Parker

**Affiliations:** 1 School of Water, Energy and Environment, Cranfield University, Cranfield, United Kingdom; 2 School of Civil Engineering, University of Leeds, United Kingdom; 3 Department of Global Development, Cornell University, Ithaca, New York, United Kingdom; 4 Bangor Business School, Bangor University, Bangor, Gwynedd, United Kingdom; 5 School of Environmental and Natural Sciences, Bangor University, Bangor, Gwynedd, United Kingdom; 6 Department of Management Science, Pontificia Universidad Católica del Perú, Perú; 7 Instituto de Estudios Peruanos, Perú; 8 Net Zero and Resilient Farming, Rothamsted Research, Harpenden, Hertfordshire, United Kingdom; 9 Department of Political Studies, University of the Western Cape, South Africa; 10 School of Population and Global Health, The University of Western Australia, Crawley, Western Australia, Australia; 11 Meru University of Science and Technology, Kenya; 12 Sanima, Av. Grau 629, Barranco, Lima, Perú; 13 School of Politics and International Studies, University of Leeds, United Kingdom; 14 Department of Landscape Architecture and the Environmental Studies Program, University of Oregon, Eugene, Oregon, United States of America; 15 Environmental Studies Program and Department of Philosophy, University of Oregon, Eugene, Oregon, United States of America; Escola de Engenharia de São Carlos da Universidade de São Paulo: Universidade de Sao Paulo Escola de Engenharia de Sao Carlos, BRAZIL

## Abstract

Hundreds of millions of people living in urban informal settlements rely on irregular and unsafe water supply and sanitation services. To meet their needs, they must navigate fragmented service delivery environments and use multiple different water and sanitation facilities. Using high-frequency longitudinal survey data from three informal settlements in Kenya, Peru and South Africa, we document the variability in water supply and sanitation service access. Across the year-long study period, 62–73% of respondents across all contexts changed their primary toilet, with 10–27% reporting five or more different primary facilities, with similar variability in water access. High levels of disruption were reported, with issues related to crowding/queuing, breakdowns, and physical barriers disrupting accessibility all contributing to the churn of services. To explain the results, we develop the concept of a “service ecosystem” to describe how people living in urban informal settlements rely on multiple water supply and sanitation services simultaneously and how these patterns of access shift over time. Using James C Scott’s theory of legibility, we argue that this irregularity means these service ecosystems are largely illegible within formal monitoring frameworks, that typically categorise households by a primary service. This leads to an information deficit for policymakers and practitioners who have a mandate to improve services in these environments. We further develop the implications of service ecosystems by calling for policymakers and service providers to recognise and support a diversity of service systems which have sufficient redundancy between them to meet the needs of populations, at least until broader structural reforms can address the underlying challenges in these settings.

## 1. Introduction

Urban informal settlements are characterised by systemic failures in water supply and sanitation service delivery. Over 1 billion people living in these settlements are routinely excluded from the core infrastructure networks of municipal service providers, particularly sewers and piped water networks, and instead become reliant on a network of off-grid household and communal systems that range in levels of formality and quality [1–3]. This leads to a fragmented service delivery environment whereby residents employ diverse and adaptive coping mechanisms – strategies and practices to mitigate service inadequacies – to meet needs around water supply and sanitation across multiple facilities [4–6].

Studies have begun to unpack these coping mechanisms showing that people access different water supply and sanitation services over time, with both diurnal variations, meaning different systems are accessible across a 24-hour period [7], as well as seasonal patterns as facility accessibility changes across the year [8]. However, these variations are hard to monitor using conventional methods. Beyond these regularised patterns of access, there are various ad hoc factors that can change the accessibility of water supply and sanitation services in informal settlements, such as poor maintenance of facilities [9], personal perceptions of safety and risk [2,10], and a tapestry of changing access rights for communal systems [11]. Taken together, this means water supply and sanitation access in urban informal settlements is typically characterised by uncertainty and change, and that there is often a misrepresentation of access in service level assessments for informal settlements [12].

There are attempts to adapt modelling approaches and sensor-based technologies to address these tensions and improve understanding of water and sanitation service dynamics in informal urban settlements. Passive Latrine Use Monitors [13] and modular water-use sensors [14] have provided real-time data on toilet usage and water consumptions, to help target sanitation facility emptying schedules and support more data-driven planning. Moreover, modellers have focused on optimising logistics for facility servicing [15] and improving decision-making about types of service delivery models and systems [16]. These approaches are often predicated on the reality of deep uncertainty regarding service delivery arrangements in informal urban settlements and there remains large gaps in understanding around the basics of water supply and sanitation provision in these contexts [17].

Both Sinharoy et al. [18] and Tumwebaze et al. [19] identify a lack of appropriate information on the reality of urban informal services restricting the development of appropriate WASH policy and interventions. It is assumed that better evidence here can inform governments, service providers and others with policy and service design; this includes helping service providers to better target populations or times with the lowest provision. This has value for the development of bespoke off-grid service delivery models, such as container-based sanitation (CBS) whereby toilets with sealable containers and relatively low infrastructural footprints are provided at the household, so that the waste can be regularly collected for treatment offsite [7]. Similarly, it can inform ‘pro-poor’ water supply arrangements like prepaid water meters that enable users to purchase water from communal water points using prepaid cards, tokens or mobile payment systems [20,21]. Improved understanding of variability might in theory also inform policymakers and local authorities with planning processes related to the full range of water supply and sanitation systems a population relies on.

Responding to these issues, this paper is guided by a core research question: How often do people change services to cope with inadequate water and sanitation services in urban informal environments? To answer this, we draw on novel high-frequency longitudinal survey datasets from informal settlements across three countries – Kenya, Peru and South Africa. To make sense of the findings and consider their wider implications, the research draws on the anthropological theory of legibility as an interpretative lens. The concept of legibility comes from the work of James C Scott [22] with the core thesis being that states attempt to make society “legible” — that is, understandable and governable — by simplifying and standardising diverse social practices into formats that can be easily managed and controlled. Typical examples include the development of property and land ownership registries which can often involve reducing the complexity of local realities to fit within legal and administrative categories, thereby making them more governable [23].

Central to our argument is that the variability and adaptability in water supply and sanitation service access in informal settlements make these contexts particularly “illegible” to formal governance systems that draw on standardised, codified information about water supply and sanitation to inform decisions-making. This creates the conditions for poor policy and inappropriate service delivery systems to develop. Our data helps us to understand the implications of this variability for the design and management of service delivery systems, and how national and international agencies can improve the monitoring of service levels in such contexts. The next section of the paper further explains the theoretical concept of legibility and its relevance for this paper, before we proceed to the methods, results and discussions.

## 2. Legibility of water supply and sanitation in informal settlements

In the classic work on legibility, James C Scott ([22] p. 2) explains “[i]n each case, officials took exceptionally complex, illegible, and local social practices, such as land tenure customs or naming customs, and created a standard grid whereby it could be centrally recorded and monitored”. This process of simplification is essential for states to govern territories and populations, but it also comes with risks [22]. The abstraction inherent in making society legible often leads to misrepresentation and oversimplification, particularly in complex environments like informal urban settlements [24], where the everyday realities of service access can defy the categories imposed by state actors [25].

To explain this process, Scott distinguishes between two types of knowledge: epistemic and metis knowledge [22]. Epistemic knowledge refers to the formalised, abstract, and often codified systems of knowledge that governments and other institutional actors use to classify and manage populations. In the realm of water supply and sanitation, epistemic knowledge manifests in the use of standardised service ladders by organisations such as the WHO-UNICEF Joint Monitoring Programme (JMP) [26], which is responsible for global monitoring of the water supply and sanitation indicators in the Sustainable Development Goals. These ladders categorise service levels into clear benchmarks such as “limited,” “basic,” and “safely managed,” allowing for comparative metrics across contexts, which are essential for global policy monitoring as well as useful for national governments in benchmarking their progress.

By contrast, metis knowledge is experiential and context-specific. It is practical knowledge, shaped by the particularities of an environment or situation [22]. In urban informal settlements, we argue that metis knowledge encompasses the nuanced, everyday practices that residents use to cope with the inadequacies and unreliability of water supply and sanitation services, including regularly changing services. This could include knowing which sanitation or water sources are safe or affordable at different times of day, understanding informal rules around the use of communal toilets, or adapting strategies to avoid health risks associated with inadequate facilities [2,11]. Scott’s argument suggests that when states and formal governance actors rely too heavily on epistemic knowledge and neglect metis knowledge, their policies and actions often fail to address the real needs of the populations they are meant to serve [22].

This analysis shares parallel with wider anthropological work on state and development policy failure. Scholars such as Ferguson [27] and Murray Li [28] draw on the Foucaldian concept of biopower in critiquing the failure of development projects and government policies that seek to govern the health and wellbeing of populations but do not account for complex, localised realities on the ground. In both cases, a tension arises between the necessity of legibility for state functioning and the risk of over-simplification, which we argue is particularly high in urban informal environments.

Yet, there is a deeper tension at play: legibility carries with it a responsibility to act [22]. The very process of making something legible to the state—mapping it, documenting it, incorporating it into formal systems—creates a duty for the state to intervene [23]. This is particularly relevant in the context of informal settlements, where limited infrastructure and service provision place significant burdens on residents. States may deliberately choose not to make certain spaces fully legible. By avoiding the creation of detailed, granular knowledge of certain populations or areas, states can avoid responsibility for addressing the needs of those populations, particularly when they lack the capacity or political will to provide adequate services. In this way, legibility is not only a tool of governance but also a strategic choice that can serve to obscure inequalities and injustices.

Ultimately, the concept of legibility highlights the risks of overlooking the complexities of informal settlements in water and sanitation policy and planning. By drawing on both epistemic and metis knowledge, policymakers and practitioners can develop a more nuanced understanding of service access in these settings, leading to more effective and equitable interventions. For example, while container-based sanitation (CBS) or prepaid water meters might offer innovative solutions, their success depends on understanding the specific ways in which these services interact with existing informal practices and coping mechanisms which are largely obscured within official data [7,29]. This study, by using high-frequency longitudinal data, aims to – at least partially – bridge this gap by providing a detailed account of the variability and adaptability of water and sanitation access in informal settlements, thereby contributing to a more legible understanding of these environments for policy and practice.

## 3. Methods

### 3.1 Study area and background

This work is part of a wider project called “Scaling-up Off-Grid Sanitation”, which aimed to understand the scalability of container-based sanitation (CBS) in informal settlements in Nairobi (Kenya), Lima (Peru), and Cape Town (South Africa). These cities exhibit distinct historical and institutional contexts that have shaped their water supply and sanitation landscapes. In Cape Town, a government run approach predominates, with the City of Cape Town providing basic sanitation services directly to informal settlements [30]. In contrast, service delivery in informal settlements in Lima and Nairobi is more fragmented, with non-governmental organizations (NGOs) playing a significant role in service provision [31]. In all three contexts, alternative sanitation solutions, including CBS, are being implemented, but the management models differ: government-led in Cape Town and NGO-led in Lima and Nairobi. The study focused on three specific settlements: BM Section of Khayelitsha (Cape Town), Pamplona Alta (Lima), and Mukuru kwa Reuben (Nairobi). These were selected as settlements where CBS was being implemented but they are broadly representative of informal settlements within these cities.

### 3.2 Study design and data collection methods

To capture the dynamic nature of water and sanitation access in these informal settlements, we conducted a high-frequency longitudinal smartphone survey. The first survey was submitted on 10^th^ May 2022 with slightly staggered start dates in each country, and a finish date ensuring that 12-months of data was collected in each city.

Participants were recruited through a multi-stage process. A gate keeper in each country was asked to select 150 CBS users. Fifty respondents were randomly selected from this list. These 50 households each selected 3 households near them with similar characteristics, except that they did not use CBS, and from each of these lists one household was randomly selected. Whilst the wider study was focused on comparing outcomes between CBS and non-CBS users, in this paper we focus on the overall patterns of water and sanitation access within the communities and therefore present data at the whole sample level. A fuller description of participant selection and sampling is provided in Lewis et al (2024).

A total of 310 participants from the three settlements took part in the survey selected. Participants responded to weekly questionnaires administered via ODK software and the Data Exchange app [32]. They were incentivised with mobile phone ownership and talk time. Incentives differed slightly between country; more details can be found in [32]. The survey covered a wide range of topics, including sanitation and water access, well-being, and demographics. This paper focuses on questions related to the variability in water supply and sanitation access (see S1 Table). To ensure comparability, we utilised the WHO-UNICEF Joint Monitoring Programme (JMP) harmonised questions for classifying water and sanitation access while introducing new questions to capture specific barriers that participants faced in accessing these services. The innovative aspect of our approach was to use these questions in a high frequency longitudinal survey, therefore allowing us to understand changes over time at a much higher resolution than conventional monitoring and studies.

The local research teams were trained in the use of the ODK methods by members of the research team that had used them in multiple similar projects [32]. These training videos and related set of supporting resources are available for wider use [32]. We then facilitated training workshops to familiarise participants with the ODK platform and questions being asked of them. Local teams then offered continued technical support through monthly workshops, phone calls, or WhatsApp, as needed.

### 3.3 Data management and analysis

The data collected throughout the year were decrypted, cleaned, and transformed for analysis. As set out in the methods paper [33], we removed invalid entries and used Python and R programming languages to perform data cleaning and analysis. Descriptive analysis of the data and one-way ANOVA were performed using the software JPM Pro 17 [34]. Violin plots were generated using the seaborn open-access library in Python [35] whilst the other plots were developed using R Studio [36]. The analysis focuses on the number and type of toilets and water sources participants report using over the time period, as well as problems the participants reported that prevented them from accessing services. The temporal changes in these variables enabled us to quantitatively describe the extent and patterns of coping strategies over time and compare them across contexts. The theoretical concept of legibility was not used as an analytical framework, rather it is introduced as an interpretative lens to understand and theorise the results we found.

### 3.4 Ethical considerations

All survey participants signed a consent form to take part in the studies. A sample consent form can be found in the reshare data repository [33]. Ethical approval across the three countries was obtained from the Bangor University College of Environmental Sciences and Engineering Ethics Committee Approval Number: COESE2021SW01A as well as from nationally-based institutional review boards (National Commission for Science, Technology and Innovation Kenya: NACOSTI/P/21/11328; University of Western Cape, South Africa; HS20/8/1; Vicerrectorado de Investigacion 033–2021-CEI-CCSSHHyAA/PUCP, Peru).

## 4. Results

**Fig 1:**
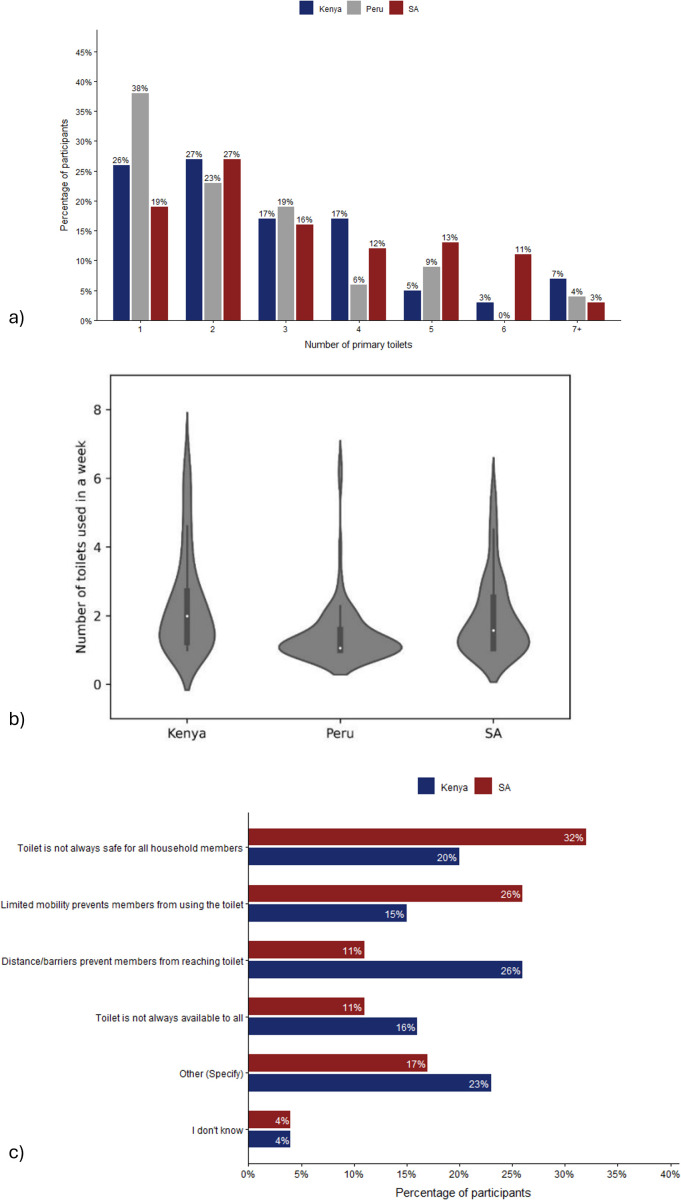
Overview of the variability in toilet use across the survey time. **a)** Number of primary toilets reported across the survey. **b)** Violin plot of the mean number of toilets people in each household used each week. **c)** Main reasons that a household cannot access a toilet during the day or night (Data: Non-CBS users from Kenya and South Africa only; there was too few responses to this question in Peru to analyse this question). NB: SA = South Africa.

Our analysis reveals a high degree of temporal variability in sanitation access across the three settlements, with water supply also showing variability, but to a lesser extent. A large proportion of respondents changed their primary toilet over the study period: in Peru, 62% of respondents reported changing their primary toilet, compared to 73% in both Kenya and South Africa. A substantial minority of respondents report a very high degree of variability by reporting five or more different primary toilets during the survey period; 27% in South Africa, 15% in Kenya and 13% in Peru (Fig 1a).

When examining toilet use over a shorter time frame of a single week (Fig 1b), the data show that respondents in Kenya had the highest mean number of toilets used per week (2.34), followed by South Africa (1.96) and Peru (1.44) (Fig 1b). Further analysis of daily and nightly access to toilets shows that various factors influenced the ability to use a facility. In Kenya, 18% of responses indicated that primary toilets were not always accessible during the day or night, with similar patterns observed in South Africa (26%) but much lower in Peru (1%). Common issues included physical barriers, safety concerns, and facilities being closed at night (Fig 1c).

**Fig 2:**
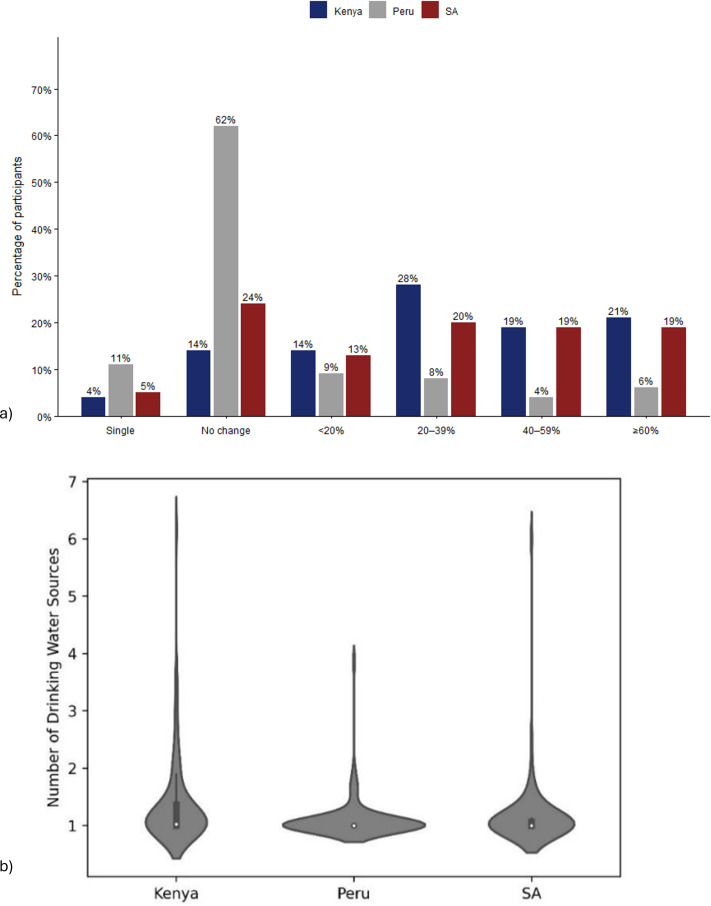
Overview of the variability in the sources of drinking water used by participants. **a)** Percentage of weeks that participants changed main source of drinking waters **b)** Violin plot of the mean number of drinking water sources people in each household used each week. NB: SA = South Africa.

**Fig 3:**
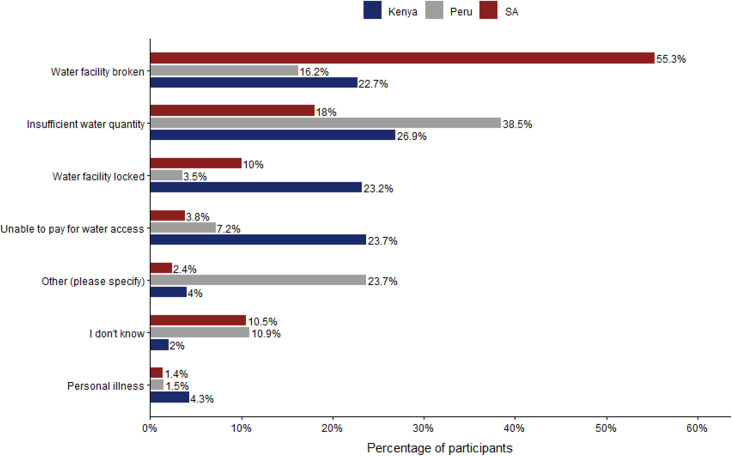
Difficulties faced by households in getting drinking water from preferred sources. NB: SA = South Africa.

The survey data also reveal variability in water source use across the settlements. While most participants in Peru (62%) did not change their main water source, only 24% of participants in Kenya and South Africa reported similar consistency (Fig 2a). This suggests a more dynamic water access environment in Kenya and South Africa, where households frequently adapt their water sources in response to changing conditions such as queuing, breakdowns and changes in the quality and quantity of water available. On average, households in Kenya reported using a greater number of drinking water sources weekly (mean: 1.35) compared to those in Peru (mean: 1.10) and South Africa (mean: 1.16) (Fig 3b), although these averages were lower than for the corresponding number of primary toilets.

As illustrated in Fig 3, even when they had access, 55%, 25% and 42% of respondents from Kenya, Peru and South Africa, respectively, suggested that they had difficulties in getting water for drinking from their preferred sources during the survey period. The most frequent issues were crowding or queuing at water facility (31%) and insufficient water quantity (15%) in Kenya, insufficient water quantity (28%) and water quality issues (22%) in Peru, and water facility being broken (35%) and crowding or queuing at water facility (23%) in South Africa. This indicates that contextual conditions influence the particularities of disruption, but that service disruption in general is a widely experienced issue.

**Fig 4:**
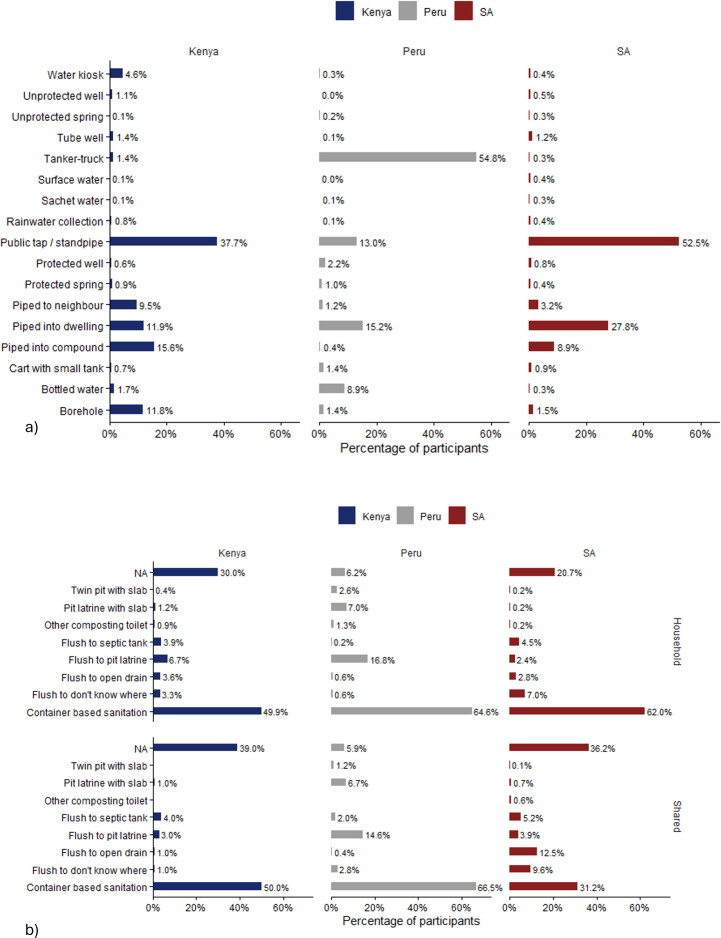
a) household reported sources for drinking water b) household reported sanitation facility types. NB: SA = South Africa.

Fig 4 shows an overview of the and sources of drinking water (Fig 4a) and type of sanitation (Fig 4a) that respondents in each location relied upon across the study period, which highlights the diversity of facilities they used. For sanitation, unsurprisingly given the purposive sampling in this study (i.e., that half the sample were recruited because they were CBS users at the outset of the study), around a half or more of total responses were for CBS, either as a household or shared facility. However, there were eight other types of sanitation systems reported across the study. For water supply, tanker-trucks is the most frequent drinking water source in Peru (54%), whereas public tap/standpipe is the most frequent drinking water source in Kenya (37%) and South Africa (52%). In Kenya there was the highest diversity, with five types of water sources – public taps, piped to neighbour, piped into dwelling, piped into compound and borehole water – being reported by around 10% or more of the population. Whilst, in Peru, there were four sources at this level – tanker trucks, public taps, piped into dwelling and bottled water, and in South Africa three sources – public tap, piped into dwelling and piped into compound.

## 5. Discussion

This paper for the first time presents data on the daily and annual variation in sanitation and water services accessed by people living in informal settlements. Respondents across all contexts reported multiple different primary toilets across the study period. These findings support the concept of “sanitation stacking” [37], a phenomena previously identified in rural Indian contexts whereby households use multiple sanitation systems simultaneously to meet their needs. Similarly, most respondents changed their primary water supply source throughout the study period, although these changes were less frequent than with sanitation. Overall, these results demonstrate that residents in urban informal settlements draw on a range of mostly off-grid water sources and sanitation facilities to overcome the challenges of inconsistent service provision. We argue that, similarly to the examples of “inlegibility” described by Scott [38], this complexity is mostly illegible within formal monitoring systems like the WHO-UNICEF JMP service ladders, which typically categorise households based on their “primary” system without accounting for this wider “service ecosystem”. This is partly unavoidable as formal governance requires ‘aggregatable’ categories – rooted in epistemic knowledge – that can be widely applied, and this means they cannot capture the fluidity of local realities.

It is important to recognise that whilst indicators have value for measuring outcomes, they should not be mistaken for the ultimate goals of the sector. Urban municipalities and utilities often have much fuller visions for sector-wide transformation, with multiple, complex objectives. The findings from this paper reinforce sectoral goals that reflect area-wide service thinking, such as articulated in planning approaches such as City-Wide Inclusive Sanitation (CWIS), that emphasises the importance of recognising the range of systems and services which meet a city’s sanitation needs [39]. However, we must recognise that the rhetorical potential of CWIS is rarely fulfilled in terms of inclusive services [40] and the conventional interpretation of CWIS is to think about how networked sewers and on-site sanitation systems together meet the needs of the population. The findings in this paper support a more dynamic view of a service ecosystem in which single households rely on a bundle of services that meet different needs over time and between household members.

To develop this theme and help identify actionable points for policy, we provide a tentative definition of a “service ecosystem”: the interconnected network of diverse services, systems, and actors that collectively meet the needs of individuals or communities, particularly in urban informal settlements where centralised infrastructure is absent or unreliable. In this context, we argue that certain service ecosystem attributes are important for authorities to focus on. These include patterns of “substitution”, which is how individuals replace or opt for alternative services, due to personal circumstances or service constraints, such as reliability and accessibility. Relatedly, the “redundancy” in the service ecosystem, which involves the overlapping capacity of water supply and sanitation systems, recognising regular failures and highly variable intersectional needs. Cutting across these, our results suggest there needs to be a high sensitivity to temporal dynamics, with needs and service availability changing throughout a 24-hour period, as well as between seasons. Urban municipalities and service providers therefore need to find ways to support and deliver this bundle of services, seek to understand substitution dynamics, and ensure redundancy across services.

Making this recommendation, we accept that many of the problems this study engages with would disappear if higher levels of service were provided, including piped water and quality household-level sanitation, although operational issues can still impact services at higher levels [41]. An example is the challenge of people meeting safely managed sanitation needs at night. However, given the limited feasibility of achieving universal access to such household services in the short term in many urban informal settlements, the type of nuanced knowledge on the frequency of service changes over time can guide intermediate improvements. For example, improving the design of business models and subsidies for off-grid solutions like CBS can make significant progress in the near term, while structural reforms for more transformative progress are pursued in parallel.

So, whilst we strongly believe that improved monitoring of services will not resolve the deeper structural barriers that hinder progress in informal settlements, we do argue that improving how we monitor services in urban informal settlements is still important. Based on current global trends, people living in urban informal settlements will become a proportionally higher percentage of the global population with inadequate water supply and sanitation services [26]. This means developing monitoring systems that reflect the needs of these populations is important to enhance accountability and guide long-term strategies. The concept of a “service ecosystem” indicates that urban informal settlements operate with fluid and adaptive approaches to service use that are not well captured by static indicators that emphasize a single “primary” service.

Building on these ideas, and reflecting wider sectoral consensus, the recent WHO [42] guidelines for managing small water supply systems advocates for a risk-based approach to monitoring. This means when faced with limited resource and capacity for monitoring, there is need for systematic prioritisation to monitor where risks are highest for public health. Those guidelines are generally referring to situations whereby a service provider or other agency may focus water quality monitoring on systems with known contamination risks, whilst conducting less frequent tests at lower risk sites. In an analogous way, we can envision a monitoring approach for urban informal settlements that emphasises highest risk situations, such as assessing the redundancy of services at nighttime, over primary service systems. More focused area-based monitoring could include indicators for service preferences, and the extent to which households and individuals use multiple services simultaneously. This would allow for a more dynamic understanding of how residents of urban informal settlements meet their water and sanitation needs, even in the face of infrastructural constraints.

This study focuses on improving the legibility of water supply and sanitation in urban informal settlements. It is based on quantitative survey results which are not conventionally amendable for studying hidden, informal behaviours. However, the use of this approach via a high-frequency mobile enabled study is novel and allows us to assess reported changes in access and practices over time in a novel way. Despite this, the survey still had some limitations. We followed the WHO-UNICEF JMP approach for household access [26], but we did not assess water quality, limiting our ability to evaluate health risks associated with different water sources. Additionally, questions about sanitation system emptying for non-CBS users were not included, restricting our understanding of the complete sanitation service chain, particularly concerning the safe management of waste. Another limitation is the reliance on self-reported data, which may introduce recall bias, especially when asking participants to account for frequent shifts in service usage – although recall biases may be lower as survey frequency increases [43]. Additionally, relying on respondents to select water supply and sanitation service types can lead to response bias when respondents do not accurately interpret the categories of systems they use (e.g., reporting a steel drum as a ‘septic tank’). The use of the training workshops and on-going participant support that allowed respondents to regularly ask questions of the research teams, were designed to reduce this bias. Data could have been analysed according to seasons but each city has its own seasonal pattern typology (summer vs winter, wet seasons vs dry season) so comparison between the cities would not have been robust.

Despite these limitations, the high-frequency longitudinal design enabled the capture of temporal variations in service access, offering a more comprehensive picture than traditional quantitative-based cross-sectional studies. Coupling the work with qualitative insights could have added more explanatory insight to the study and, in parallel, we conducted qualitative studies which dug deeper into the reasons behind people’s choices and mitigate these limitations to some extent, although these had distinct analytical approaches and it was deemed not feasible to include in a single paper. However, these largely complement and strengthen the points made here. For example, in Nairobi the cultural beliefs and taboos surrounding sanitation have made it a taboo topic and this has hindered effective sanitation initiatives leaving piecemeal provision [44] in Cape Town, our qualitative work shows how shared facilities are often locked by one individual, preventing other households from accessing them [45,46], even when they nominally have the right to access the facility. Such qualitative vignettes into the lived experience of water supply and sanitation access in informal settlements reveal the deeply contextual – *metis knowledge-based* – reality of urban life and provide complementary insights into the quantitative data and theoretical discussions provided in this paper.

## 6. Conclusion

As far as we can assess, this study is the first to use high frequency survey data to show the extent to which water and sanitation access in urban informal settlements are highly variable, with households frequently relying on multiple systems to meet their needs and facing high levels of disruption in the services they can access. We propose the idea of a “service ecosystem” to describe this and, drawing on James C. Scott’s concept of legibility [22], we argue that such service ecosystems are largely illegible to formal monitoring systems. Future research should therefore focus on developing a more nuanced understanding of these service ecosystems and assess their consistency and dynamism across more settings, with a view to supporting the development of appropriate monitoring frameworks, providing data to aligned modelling studies, and providing evidence that can underpin policy that is more sensitive to these complex realities.

At the same time, we must acknowledge that granular knowledge and improved monitoring cannot solve the deeper structural issues that underpin poor service provision in most urban informal settlements. Land tenure insecurity, political exclusion, and financial constraints all limit the ability of governments and utilities to provide higher levels of service [47]. Addressing these structural barriers requires long-term, systemic reforms that go beyond the scope of any single intervention. Meaningful progress will depend on developing comprehensive sectoral goals and actions that address the root causes of inadequate services. Monitoring can support this process, but it must remain one part of a much larger strategy for transformative change in the urban WASH sector.

## Supporting information

S1 File Tables S1-S6. (DOCX)

S2 File Inclusivity-in-global-research-questionnaire. (PDF)
