# Supplementary material for: Beyond the grid: Navigating water supply and sanitation service ecosystems in informal settlements
Source: PLoS One. 2026 Mar 13;21(3):e0342657. doi: 10.1371/journal.pone.0342657 (PMC12987421; doi:10.1371/journal.pone.0342657)
Supplement: S1 File — (DOCX) [file pone.0342657.s001.docx]

**Supplementary material:** Tables S1-S6.

Table S1. Survey questions and detail of responses

| **Question** | **Responses** |
| --- | --- |
| **Sanitation access** |  |
| How many toilets did people in your household regularly use this week? | Integer |
| Do you share your primary toilet with others who are not members of your household? | Yes  No  I don’t know |
| Which one would you consider your households primary toilet? | Container based sanitation  Flush to piped sewer system  Flush to septic tank  Flush to pit latrine  Flush to open drain  Flush to don't know where  Pit latrine with slab  Pit latrine without slab/ Open pit  Twin pit with slab  Twin pit without slab  Other composting toilet  Bucket (informal)  Hanging toilet/ hanging latrine  No facility / Bush / Field |
| Where did you use the bathroom this week? | A neighbor's house  School  A public toilet  I don't know  My workplace  Business  Church  Outside (in open spaces)  Inside my home (in a fixed location)  Inside my home (stored and brought out for use),  Outside my home (in a separate structure) |
| Is everyone in the household able to access and use the primary toilet at all times of day and night? | Yes  No  I don't know |
| What was the main reason that household members were unable to use the primary toilet at all times of the day or night? | Limited mobility prevents members from using the toilet  Distance/barriers prevent members from reaching the toilet  Toilet is not always available to all household members  Toilet is not always safe for all household member to use  Other (Specify)  I don't know |
| Did you have any problems with the primary toilet itself this week? | Not at all a problem  Minor problem  Moderate problem  Serious problem  I don't know |
| [If there was a problem] What kind of problems? (multiple-choice question) | Smelled bad  Needs emptying  Needs repairs  Other  I don't know |
| **Water access** |  |
| What is the main source of drinking water for members of your household?  Which of these sources for drinking water have you relied upon since we last spoke? (multiple-choice question) | Piped into dwelling  Piped into compound, yard or plot  Piped to neighbour  Public tap / standpipe  Tube Well  Borehole  Protected well  Unprotected well  Protected spring  Unprotected spring  Rainwater collection  Tanker-truck  Cart with small tank / drum  Water kiosk  Surface water (river, stream, dam, lake, pond, canal, irrigation channel)  Bottled water  Sachet water  Other (specify)  I don't know |
| How long does it take to go to the place where you collect your main source of drinking water from, get water, and come back? (in minutes) | Integer |
| Did you have any difficulty in getting water for drinking from your preferred sources? | Yes  No  I don't know |
| [if yes] What did you find constraining? | Water facility broken  Crowding or queuing at water facility  Water facility locked  Unable to pay for water access  Water quality issue  Insufficient water quantity  Personal illness  Other (please specify)  I don't know |

Table S2. Descriptive statistics of the average number of weekly toilets reported across the survey time

| **Statistics** | **Kenya** | **Peru** | **South Africa** |
| --- | --- | --- | --- |
| count | 108 | 94 | 93 |
| mean | 2.34 | 1.44 | 1.96 |
| standard deviation | 1.48 | 0.88 | 1.15 |
| minimum | 1.00 | 1.00 | 1.00 |
| median | 1.99 | 1.06 | 1.57 |
| maximum | 6.75 | 6.38 | 5.67 |

Table S3. Descriptive statistics of the number of primary toilets reported across the survey time: a) Kenya, b) Peru, c) South Africa

a)

| **Level** | **Number** | **Mean** | **Std Dev** | **Std Err Mean** | **Lower 95%** | **Upper 95%** | **Std Dev Lower 95%** | **Std Dev Upper 95%** |
| --- | --- | --- | --- | --- | --- | --- | --- | --- |
| CBS | 1716 | 2.68 | 2.70 | 0.06 | 2.55 | 2.80 | 2.62 | 2.80 |
| NU | 1738 | 3.08 | 4.14 | 0.10 | 2.88 | 3.27 | 4.00 | 4.28 |

Difference highly significant, p<0.001

b)

| **Level** | **Number** | **Mean** | **Std Dev** | **Std Err Mean** | **Lower 95%** | **Upper 95%** | **Std Dev Lower 95%** | **Std Dev Upper 95%** |
| --- | --- | --- | --- | --- | --- | --- | --- | --- |
| CBS | 806 | 2.24 | 3.64 | 0.13 | 1.99 | 2.50 | 3.48 | 3.83 |
| NU | 1878 | 1.32 | 1.10 | 0.03 | 1.27 | 1.37 | 1.07 | 1.14 |

Difference highly significant, p<0.001

c)

| **Level** | **Number** | **Mean** | **Std Dev** | **Std Err Mean** | **Lower 95%** | **Upper 95%** | **Std Dev Lower 95%** | **Std Dev Upper 95%** |
| --- | --- | --- | --- | --- | --- | --- | --- | --- |
| CBS | 954 | 2.00 | 2.33 | 0.08 | 1.85 | 2.15 | 2.23 | 2.45 |
| NU | 698 | 2.99 | 3.36 | 0.12 | 2.74 | 3.24 | 3.19 | 3.55 |

Difference highly significant, p<0.001

Table S4. Distribution of locations of toilets used weekly a) CBS users, b) non-users

a)

| Columns by Categories | Kenya, % of all responses received |  | Peru, % of all responses received |  | South Africa, % of all responses received |  |
| --- | --- | --- | --- | --- | --- | --- |
| home fixed | 29.93% |  | 50.73% |  | 19.04% |  |
| public toilet | 13.82% |  | 7.19% |  | 24.01% |  |
| outside separate structure | 17.66% |  | 2.80% |  | 21.52% |  |
| at work | 5.72% |  | 29.56% |  | 5.46% |  |
| neighbour's house | 12.10% |  | 4.39% |  | 8.44% |  |
| outside open space | 7.28% |  | 0.80% |  | 6.62% |  |
| at school | 4.82% |  | 0.80% |  | 3.81% |  |
| at home stored | 3.27% |  | 2.00% |  | 6.29% |  |
| church | 2.21% |  | 0.00% |  | 1.49% |  |
| business | 2.21% |  | 0.00% |  | 0.83% |  |
| don't know | 0.98% |  | 1.60% |  | 2.48% |  |
| All responses | 100.00% |  | 100% |  | 100.00% |  |

b)

| Columns by Categories | Kenya, % of all responses received |  | Peru, % of all responses received |  | South Africa, % of all responses received |  |
| --- | --- | --- | --- | --- | --- | --- |
| home fixed | 32.43% |  | 37.26% |  | 6.14% |  |
| public toilet | 17.36% |  | 18.20% |  | 44.59% |  |
| outside separate structure | 15.61% |  | 8.74% |  | 4.95% |  |
| at work | 12.41% |  | 15.77% |  | 11.49% |  |
| neighbour's house | 10.89% |  | 5.73% |  | 9.51% |  |
| outside open space | 2.44% | 0.40% | 2.44% | 0.21% | 5.75% | 0.36% |
| at school | 2.06% | 0.34% | 4.16% | 0.36% | 4.95% | 0.31% |
| at home stored | 1.67% | 0.28% | 2.29% | 0.20% | 4.36% | 0.28% |
| church | 3.43% | 0.57% | 1.29% | 0.11% | 6.94% | 0.44% |
| business | 1.37% | 0.23% | 2.29% | 0.20% | 0.99% | 0.06% |
| don't know | 0.38% | 0.06% | 1.86% | 0.16% | 0.40% | 0.03% |
| All responses | 100% | 16.53% | 100% | 8.78% | 100% | 6.35% |

Table S5. Descriptive statistics of the average number of main drinking sources used across the survey time

| **Statistics** | **Kenya** | **Peru** | **South Africa** |
| --- | --- | --- | --- |
| count | 106 | 89 | 88 |
| mean | 1.35 | 1.10 | 1.16 |
| standard deviation | 0.73 | 0.35 | 0.58 |
| minimum | 1.00 | 1.00 | 1.00 |
| median | 1.03 | 1.00 | 1.00 |
| maximum | 6.16 | 3.85 | 6.00 |

Table S6. Descriptive statistics of the average length of time it takes for each household to collect water (round trip including queue time) reported across the survey time

| **Statistics** | **Kenya** | **Peru** | **South Africa** |
| --- | --- | --- | --- |
| count | 105 | 89 | 88 |
| mean | 9.91 | 13.06 | 5.56 |
| standard deviation | 9.09 | 14.08 | 5.70 |
| minimum | 1.58 | 0.00 | 0.00 |
| median | 6.38 | 6.67 | 3.67 |
| maximum | 45.00 | 90.00 | 27.50 |
