# Supplementary material for: Beyond the grid: Navigating water supply and sanitation service ecosystems in informal settlements
Source: PLoS One. 2026 Mar 13;21(3):e0342657. doi: 10.1371/journal.pone.0342657 (PMC12987421; doi:10.1371/journal.pone.0342657)
Supplement: S2 File — (PDF) [file pone.0342657.s002.pdf]

Inclusivity in global research

PLOS' policy on inclusivity in global research aims to improve transparency in the reporting of research performed outside of researchers' own country or community and ensures that PLOS publications reporting global research adhere to high standards for research ethics and authorship. Authors of relevant research articles may be asked to complete the questionnaire below, which outlines ethical, cultural, and scientific considerations specific to inclusivity in global research. This questionnaire may be requested when researchers have travelled to a different country to conduct research, if research uses samples collected in another country, research with Indigenous populations or their lands, or if research is on cultural artefacts. Researchers travelling to another country solely to use laboratory equipment will not normally be required to complete the questionnaire. However, the questionnaire can be requested at the journal's discretion for any submission – if you have been requested to complete this questionnaire by the PLOS journal you submitted to, please do so.

Please complete the questionnaire below and include this as a Supporting Information file with your manuscript. Note that if your paper is accepted for publication, this checklist will be published with your article in the supporting information files. Please ensure that you reference the checklist in the main body of your manuscript. We suggest adding a subsection 'Inclusivity in global research' to your Methods section and adding the following sentence: "Additional information regarding the ethical, cultural, and scientific considerations specific to inclusivity in global research is included in the Supporting Information (SX Checklist)"

The questions have been designed to be applicable to a wide range of study types, and there are subsections for both human subjects research and non-human subjects research. If any of the questions are not relevant to your research please mark them as "N/A" as appropriate.

Ethical considerations, permits and authorship

This section is applicable to all research types.

Provide details as to who granted permissions and/or consent for the study to take place in the Methods section of your manuscript. This should include the names of **all** ethics boards, governmental organizations, community leaders or other bodies that provided approval for the study. If individuals provided approval refer to these people by their role or title but do not list their name(s).

Reported on page number: P7: "All survey participants signed a consent form to take part in the studies. A sample consent form can be found in the reshare data repository (Lewis et al., 2024). Ethical approval across the three countries was obtained from the Bangor University College of Environmental Sciences and Engineering Ethics Committee Approval Number: COESE2021SW01A as well as from nationally-based institutional review boards (National Commission for Science, Technology and Innovation Kenya: NACOSTI/P/21/11328; University of Western Cape, South Africa; HS20/8/1; Vicerrectorado de Investigacion 033-2021-CEI-CCSSHHyAA/PUCP, Peru)."

If there were any deviations from the study protocol after approval was obtained please provide details of these changes in the Methods section of your manuscript.

Reported on page number: This study did not have a protocol deposited on a public registry as it was not a study design where this is typical (e.g. not a intervention trial or systematic review). We did not deviate from the approved protocol that was in the ethical forms.

Did this study involve local collaborators that are residents of the country where the research was conducted or members of the community studied? If you do not have any authors from said communities, please provide an explanation for this below.

Yes – authors from all countries involved in the study are involved, including Peru, South Africa and Kenya.

Everyone listed as an author should meet PLOS' criteria for authorship and all individuals who meet these criteria should be included in the author byline, rather than the acknowledgements. For further information please see the journal's Authorship Policy.

Human subjects research (e.g. health research, medical research, cross-cultural psychology)

Did you obtain written informed consent from a representative of the local community or region before the research took place? How did you establish who speaks for the community? Details of written informed consent obtained from study participants should be reported separately in the Methods section of your manuscript.

The study was based on a mobile-phone based survey and we obtained consent from every participant in the study on an individual basis, as outlined in the methods.

How did members of the local community provide input on the aims of the research investigation, its methodology, and its anticipated outcome(s)?

The study was not a participatory study in the sense that the research objectives were co-designed with the local population. We ran workshops in the community as part of the recruitment and training of participants in using the mobile-phone based survey, as we outline in the methods.

When engaging with the local community, how did you ensure that the informed consent documents and other materials could be understood by local stakeholders?

The research teams in each context – Universities from the cities where the study took place – had local knowledge, networks and spoke local languages. All survey tools and consent were translated into local languages and piloted with local communities.

Will the findings of the research be made available in an understandable format to stakeholders in the community where the study was conducted (e.g. via a presentation, summary report, copies of publications, etc.)? Please provide details of how this will be achieved.

The local research teams presented the research back to local communities in workshops at the end of the study period.

Non-human subjects research using specimens/ animals collected as part of the study, or those housed in archival collections. Examples include archaeology, paleontology, botany and zoology.

Did the permission you obtained from a local authority to perform the study include an agreement on access to outputs and benefit sharing? This may include procedures to enable fair distribution of the benefits and resources arising from the research performed. Please include any details of Prior Informed Consent and Benefit Sharing Agreements obtained. These may be required by field-specific regulations, for example the Convention on Biological Diversity (CBD) and the associated Nagoya Protocol.

N/A

If the material used in your study was imported, please A) provide the year it was imported and B) indicate whether permits were obtained to import/export the materials used, C) provide details of any permits obtained. If this information is not available, please indicate this.

N/A

If you used archival specimens, please state how the material used in your study was acquired by the institute it is held in and provide details of any permits obtained for the original excavations/ sample collection. If this information is not available, please indicate this.

N/A

How was the potential cultural significance of the materials collected in your study to local communities considered in your research design? Were Indigenous peoples and/or local researchers and institutions involved with archaeological excavations / collection of specimens? If so, please provide a description of their involvement.

N/A

If your manuscript includes photographs of human remains please indicate whether authors obtained permission from descendants or affiliated cultural communities to do so.

N/A
